# Memory Systems, the Epistemic Arrow of Time, and the Second Law

**DOI:** 10.3390/e26020170

**Published:** 2024-02-16

**Authors:** David H. Wolpert, Jens Kipper

**Affiliations:** 1 Santa Fe Institute, 1399 Hyde Park Road, Santa Fe, NM 87501, USA; 2 Philosophy Department, University of Rochester, Rochester, NY 14627, USA; jens.kipper@rochester.edu

**Keywords:** second law of thermodynamics, psychological arrow of time, Brownian bridge, memory systems, records

## Abstract

The epistemic arrow of time is the fact that our knowledge of the past seems to be both of a different kind and more detailed than our knowledge of the future. Just like with the other arrows of time, it has often been speculated that the epistemic arrow arises due to the second law of thermodynamics. In this paper, we investigate the epistemic arrow of time using a fully formal framework. We begin by defining a memory system as any physical system whose present state can provide information about the state of the external world at some time other than the present. We then identify two types of memory systems in our universe, along with an important special case of the first type, which we distinguish as a third type of memory system. We show that two of these types of memory systems are time-symmetric, able to provide knowledge about both the past and the future. However, the third type of memory systems exploits the second law of thermodynamics, at least in all of its instances in our universe that we are aware of. The result is that in our universe, this type of memory system only ever provides information about the past. We also argue that human memory is of this third type, completing the argument. We end by scrutinizing the basis of the second law itself. This uncovers a previously unappreciated formal problem for common arguments that try to derive the second law from the “Past Hypothesis”, i.e., from the claim that the very early universe was in a state of extremely low entropy. Our analysis is indebted to prior work by one of us but expands and improves upon this work in several respects.

## 1. Introduction

It seems obvious that our knowledge of the past is of a different kind and more detailed than our knowledge of the future. It is far less obvious what explains this so-called ‘epistemic arrow’ of time. As with the other arrows of time, the fact that the fundamental physical laws are time-symmetric presents a major obstacle to finding such an explanation. Many philosophers and scientists have suggested explanations that appeal to the (time-asymmetric) second law of thermodynamics, or to some more fundamental facts underlying the second law [1,2,3,4,5,6,7,8,9,10]. David Albert [11,12,13,14] and Barry Loewer [15,16,17] have developed one such account that has been particularly influential in recent years. We are sympathetic to their account, but we believe that it has a crucial gap.

Our own account is based on a formal distinction between three types of memory systems that occur in the physical universe. By ‘memory system’, we here mean any kind of physical system whose present state can provide information about the state of the external world at some time other than the present. This use of the word ‘memory’ is undoubtedly broader than the ordinary use of the term. It should thus be understood as a purely stipulative definition for the purposes of our present discussion. On the basis of our formalism, we show that physical systems exemplifying either of the first two types of memory systems can be sources of knowledge about both the past and the future. The epistemic arrow must therefore be grounded in the third type of memory systems. We argue that, plausibly, all memory systems of this type exploit a reduction in state space, which implies that the information they provide can only be of the past. Finally, we argue that human memory is of this third type. Our paper is indebted to the analysis in Wolpert [18], but expands and improves upon it in several respects.

The paper is structured as follows. In Section 2, we discuss Albert and Loewer’s account. As we argue, their explanation of the epistemic arrow does not get off the ground without the doubtful assumption that typically, the systems we have knowledge from or those we have knowledge about had a lower entropy in the past. We suggest that such an explanation should instead be based on the idea that the process of creating information involves an increase in entropy.

In Section 3, we distinguish the three different types of memory systems we find in the physical universe, and present high-level examples of each type. In Section 4, we introduce our formalism that captures the three different types of memory systems. We show that the third type is a special case of the first type of memory systems. Our investigation of how these memory systems can function reveals that one of them, namely Type-3 memory systems, can and perhaps must rely on the second law, which implies that it is time-asymmetric. In Section 5, we first discuss whether our account can capture the (putative) asymmetry of records. We then provide reasons for thinking that human memory exemplifies Type-3 memory, which means that our account is suitable for explaining the epistemic arrow of time in terms of the second law of thermodynamics. In Section 6, we discuss whether the second law itself—and hence the epistemic arrow—can be reduced to even more fundamental facts. While we remain open to this idea, we show that common arguments for the claim that the second law follows from the assumption of a low-entropy state in the very early universe are problematic. (Specifically, we describe a previously unappreciated formal problem with that argument, grounded in Markov process theory.) Finally, in Section 7, we spell out some remaining issues to be addressed by future research.

## 2. Albert and Loewer on the Asymmetry of Records

Albert and Loewer’s account is part of a highly ambitious project that aims to explain, among other things, all arrows of time. It begins, in essence, with what in the physics community has been called the argument for the “cosmological origin of the arrow of time” [19]. One of its key components is what Albert and Loewer call the “Past Hypothesis”, which is the assumption that the entropy of the very early universe was very low. They combine this assumption with the fact that the dynamical micro-physical laws are deterministic and time-symmetric, and with a “probability postulate”. The latter corresponds to the standard microcanonical ensemble from statistical physics, which follows from the maximum entropy principle of inference [20], and says that there is a uniform probability distribution over the microstates compatible with the Past Hypothesis. Together, these three components determine a probability assignment to all propositions about the history of the universe. Albert [13] calls this probability assignment the “Mentaculus”.

Albert and Loewer claim that these three components also explain the “epistemic arrow of time”, by which they mean the fact that all records are of the past. (It is worth noting that many other philosophers have also appealed to an asymmetry of records, e.g., Reichenbach [6]).) Intuitive examples of records are impact craters, footsteps on a beach, diary entries, and memory traces in the brain. Albert (Chapter 6 in [13]) calls inference procedures that use dynamical laws to evolve macroscopic information about the present forward or backward “predictions” and “retrodictions”, respectively. He states that records are those inference procedures to other times that are not predictions or retrodictions. A record is created when a recording device interacts with the external world—Albert calls this interaction a “measurement”. In typical cases, the state of the recording device then remains stable, which allows drawing inferences from its current state about the state of the external world at the time of the interaction. Albert and Loewer claim that this inference requires that the recording device is in a particular state—the “ready state”—before the interaction (see [18] for earlier work using the same terminology of “predictions” and “retrodictions”, making the same point about the stability of the recording device, using the same examples, and also highlighting the importance of what they call a “ready state”).

It thus appears that to obtain information from a record, we need to know what the ready state obtained. But knowing that, in turn, seems to require another measurement, setting up a potential infinite regress. This regress is stopped, according to Albert and Loewer, by the Past Hypothesis, which serves as the universe’s “ultimate ready state”. By conditioning on it, they claim, we can acquire knowledge of the past from records.

However, obviously, people had knowledge from records long before anyone had ever thought of the Past Hypothesis. Moreover, when we observe a record, our backward-chain of remembered measurements terminates much more recently than 13 billion years ago, the time of the initial state of the universe. Given this, how could the Past Hypothesis help us infer that our recording device was in its ready state? As Albert explains (pp. 355–357, [14]), the account is not meant to assume that knowledge from records relies on explicit inferences from the Past Hypothesis. Rather, when we observe a record, the initial low-entropy state of the universe just makes it much more likely that the recording device was in its ready state before the time of the interaction, and that fact is “hard-wired” into how we make inferences. The crucial question is thus how the Past Hypothesis licenses the inference, with high probability, that a given recording device was in its ready state before the relevant interaction took place. Albert and Loewer say surprisingly little about this issue. But they do provide some hints. For instance, Albert illustrates their account with the example of a half-melted block of ice sitting on the floor of a warm room. According to Albert, conditioned on the Past Hypothesis, it is likely that the block of ice was less melted several minutes in the past, and our inferences concerning it implicitly rely on this fact. Sean Carroll (p. 40, [1]) uses the example of a half-rotten egg to offer a very similar account of the role of the Past Hypothesis in inferences from records. He adds that, due to the thermodynamic arrow, that entropy increases in time and the egg’s current state offers us much less information about its future states than about its past states. (Notice that the block of ice and the rotting egg are examples of systems whose current state provides information *about its own state* at a different time, rather than about the external world. If one does consider such systems as records, then many records can present information about the future. For example, a gas cloud with sufficient density, mass, etc., can be predicted to form a planet. Further examples of this type are provided by other nonlinear dynamical systems with a point attractor and an associated basin of attraction. As described below, in this paper, we instead consider systems that provide information about *the state of the world external to such a system* at a different moment of time. We claim that that is one of the key features of the epistemic arrow.)

Loewer [15,16] generalizes this idea. He argues that, given the initial low-entropy state of the universe and the resulting thermodynamic arrow, information about a system’s present state constrains its past states much more than it constrains its future states. The Past Hypothesis effectively imposes a tree structure on the history of the universe, with many more branches leading to the future than to the past. According to him, this implies that, typically, observations about the present state of a system offer us more information about its past than about its future. The following quote spells out this idea:

The SM [i.e., statistical–mechanical] probability distribution embodies a way in which “the future” (i.e., the temporal direction away from the time at which PH [i.e., the Past Hypothesis] obtains) is “open” at least insofar as macro states are being considered. Since all histories must satisfy the PH, they are very constrained at one boundary condition, but there is no similar constraint at other times. It is true that (almost) all histories eventually end up in an equilibrium state (there is a time at which almost all histories are in an equilibrium state), but this is not a constraint, it is a consequence of the dynamics and the PH, and it is not very constraining (almost all states are equilibrium states). Another feature of the SM distribution when applied to the macro state of the kind of world we find ourselves in is that the macro state of the world at any time is compatible with micro states that lead to rather different macro futures. For example, conditional on the present macro state of the world, the SM probability distribution may assign substantial chances both to it raining and not raining tomorrow. On the other hand, there is typically much less branching towards the past. The reason is that the macro states that arise in our world typically contain many macroscopic signatures (i.e., macro states/events that record other macro states/events) of past events but fewer macroscopic signatures of future states/events. Newspapers are much more accurate in recording past weather than in predicting future weather. Of course, these two features of the SM distribution—that histories are very constrained at one boundary condition but not at other times and that they branch much more to the future (direction away from the PH)—are related.

(pp. 302–303, [15])

As this quote indicates, the basic idea behind Albert and Loewer’s explanation is that because there are many more high-entropy than low-entropy states, the Past Hypothesis creates an asymmetry of information: given the Past Hypothesis, there are many more “branches” from the present towards the future than towards the past. Loewer relates this asymmetry directly to the asymmetry of records, which Albert and Loewer consider to constitute the epistemic arrow of time. The examples of the half-melted ice block and the half-rotten egg illustrate this idea.

Albert and Loewer’s explanation of the epistemic arrow is suggestive, and it has been highly influential, even though it has also been much criticized (see, e.g., Earman [21], Frisch [22,23,24], Huggett [25] and Winsberg [26], pp. 420–422). Here, we highlight a lacuna in their account that, to our knowledge, has not yet been identified. This helps us formulate a general adequacy condition for an explanation of the epistemic arrow.

Albert and Loewer’s explanation is only applicable to systems that mirror the entropy gradient of the universe. This is because in their account, the temporal asymmetry of “branching”, which is supposed to explain the epistemic arrow, relies on the idea that the entropy of the systems in question is increasing. This idea is reflected in the examples of the melting ice block and the rotting egg, in which the entropy of both the objects of our knowledge, i.e., of the systems we have knowledge about, and of the recording systems is increasing. However, the epistemic arrow applies to many systems whose entropy is not increasing. For instance, we can have much more knowledge about what state a weather system was in five weeks ago than about what state it will be in five weeks from now. (We can know its past by looking at records that we made in the past of what its past state was, whereas we have no such records of what its future will be.) Of course, a weather system is an open system that takes in energy from the sun, and thus it does not violate the second law. Nevertheless, the local system itself is typically entropy-neutral. Since this is the system we observe, it is thus unclear how its present state would constrain its past state more than its future state. One might try to argue that such systems are not typical. But as the following considerations show, this position is untenable.

Since the appearance of the first cognitive systems on our planet, both the sources and the objects of their information have almost exclusively been physical systems on Earth. Despite our recent efforts to learn more about the solar system and the universe outside of it, this is still very much the case. The Earth system itself has remained far from thermodynamic equilibrium for a very long time. Roughly speaking, this is possible because Earth is an open system that takes in free (relatively low-entropy) energy from the sun and radiates away high-entropy waste heat. The entirety of the Earth system appears to be entropy-neutral—it has even been argued that its entropy has steadily decreased over the last hundreds of millions of years [27,28]. This strongly suggests that typical systems that we have information from as well as knowledge about do not exhibit an increase in entropy—there should be at least as many such systems whose entropy remains constant or is even decreasing.

At various points, Loewer adds the qualification that the relevant systems must be at least approximately thermally isolated (e.g., Loewer [15,29]). It is, of course, likely that most thermally isolated systems that we have knowledge from or about evolve towards equilibrium. But it is not apparent how this could be of help to their explanation of the epistemic arrow, since most of the systems that we have knowledge from and knowledge about are not even approximately thermally isolated. As we just saw, the Earth system as a whole falls into this category. Therefore, the Earth system does not exhibit the tree structure postulated by Loewer.

We conclude that Albert and Loewer’s explanation of the epistemic arrow is at least incomplete. As we saw, a fully adequate explanation must be compatible with the fact that the entropy of many, if not most, of the systems we have knowledge from or knowledge about is not increasing. (In fact, as we elaborate below, initializing memory systems into a ready state often involves *reducing* their entropy, sometimes even into a state of minimal entropy). Therefore, such an explanation should not appeal to the entropy gradient of the objects of our knowledge or of the recording systems. (It is, of course, open to Albert and Loewer to explain their interpretation of the epistemic arrow of time in some other way. In fact, as we discuss in Section 5, our own account, which does not rely on the assumption that the systems that we have knowledge from or about are increasing in entropy, could potentially be used to explain the asymmetry of records. But our point here is that Albert and Loewer do not provide such an explanation.)

This condition is violated in other accounts of the epistemic arrow besides that of Albert and Loewer. For example, ref. [30] presents four conditions for a system to be a memory. Unfortunately, the fourth condition states that *by definition*, memory systems cannot work unless they rely on the second law. (Specifically, it is said there that Condition 4 “is a reflection of our assumption that there exists a thermodynamic arrow of time. The question of whether a memory can record the future makes no sense otherwise, since it is the thermodynamic arrow that we use to define past and future.” But it is hard to be sure, because [30] is informal in its discussion of the implications of those four conditions, not presenting any mathematical proofs.) It is not surprising then that [30] overlooks the possibility of time-symmetric memories like those that work in computers. In contrast, our definitions of memory systems rely *exclusively* on statistical coupling between the state of the memory system now and the state of the external world (the variable that the memory system actually “remembers”) at a different time. The question of whether and what laws of physics can enable such memory systems is then *derived* rather than assumed a priori.

Summarizing, in contrast to these earlier approaches, our investigation of the epistemic arrow of time, i.e., of the asymmetry in our knowledge of the past and of the future, does not assume that this arrow is constituted by an a priori asymmetry in the objects of our knowledge or of recording systems. Instead, our investigation starts by introducing a distinction between three types of memory systems. We then provide fully formal definitions of these three types in terms of statistical relationships between different random variables, showing that they reflect three ways for information about the state of one system at one time to be conveyed to the state of another system at another time.

Importantly, two of these three types do not yield a temporal asymmetry, and thus these memory systems do *not* result in an epistemic arrow. In contrast, another type of memory system we analyze involves a special initialized state (i.e., the “ready state”). This state allows information to be conveyed from one moment to another, created by a process that increases global entropy. This kind of system thus relies on the second law of thermodynamics, just like those considered by Albert and Loewer. However, in this type of system, no assumption is made about the entropy gradient of the system it carries information about. Furthermore, the initialized state, too, need not have lower entropy than the current state. Indeed, we demonstrate that in common examples of the epistemic arrow, the initialized state has *higher* entropy than the current state. (See example of memory systems involving stones on the bottom of a cave in Section 4.)

## 3. Three Types of Memory Systems

A “memory system”, as we understand the term here, is any physical system whose state at the present time, $t_{0}$, carries information about the state of the world at time $t_{1}\neq t_{0}$, where $t_{1}$ can be either in the future or in the past. By “carry information”, we mean that due to the joint probability distribution of the state of the memory at $t_{0}$ and the state of the world at $t_{1}$, knowing the state of the memory at $t_{0}$ provides us with extra information concerning the state of the world at $t_{1}$, beyond our prior information about the state of the world at that time $t_{1}$. We are interested in the forms of such joint distributions that seem to occur in the real world.

### 3.1. Intuitive Examples of Memory Systems

To formulate this idea more carefully, we let *M* and *W* be the state spaces of a memory system and of the external world, respectively. Axiomatically, our probability distributions involve the states of *M* and *W* and the two times $t_{0}$ and $t_{1}$. In addition, below, we show that in real-world examples of memory systems, the states of *M* and/or *W* at another time $t_{2}$ may play a role, where either $t_{0}<t_{1}<t_{2}$ or $t_{0}>t_{1}>t_{2}$. Associated with the two systems M,W and the three times $t_{0},t_{1}$ and $t_{2}$, we have six jointly distributed random variables, $W_{0}$, $W_{1}$, $W_{2}$, $M_{0}$, $M_{1}$, and $M_{2}$. Our formalizations of different types of memory system specifies different properties of that joint distribution.

In this paper, we often do not discuss how we have come to know (!) that the joint probability $P(w_{0},m_{0},w_{1},m_{1},w_{2},m_{2})$ over the six random variables has those properties, or where this distribution comes from, i.e., what physical process may have been involved in its creation. Similarly, we are often very loose with the terminology and say that we “observe” the state of a variable at a particular time, as shorthand for saying that we acquire some possibly noisy information about its state. Formally, such an observation involves yet more random variables, statistically coupled with the ones described above. We ignore such variables here. (We do not mean to imply anything more than this shorthand when we use the term “observe”. In particular, we do not imply anything involving the nature of observation in quantum mechanics.)

For simplicity, we speak as though this information we acquire concerns the memory’s present state *exactly*, to infinite precision. Some might object that in the real world, infinite precision requires an infinite number of digits, to perfect accuracy, and real systems simply do not have that capability. Accordingly, typically, we implicitly assume that *M* and *W* are elements in coarse-grainings of states in some associated phase spaces. It is straightforward to extend our reasoning to accommodate noisy, imprecise information about those states rather than such coarse-graining. (See also our discussion below concerning measure-theoretic integrals.)

In some cases, the memory works by combining information about the present state of memory system *M* with information about the present state of external world *W*. We thus allow for the possibility that in addition to observing value $m_{0}$, the user of that memory system knows that $w_{0}$ falls within some particular set. We are careful not to stipulate that the user of the memory system “observes” whether that is the case; they may simply assume it. From this information about $m_{0}$ and possibly $w_{0}$, we want to draw a probabilistic inference about the state of the external world at another time, $w_{1}$.

Since the memory system’s present state should be relevant to the inference we draw, we require that its information about $w_{1}$ varies depending on the value of $M_{0}$. Physically, when this condition is satisfied, we can infer from the observed $m_{0}$ (perhaps in conjunction with some information about $w_{0}$) that *M* and *W* interacted sometime between $t_{0}$ and $t_{1}$, such that, in the course of this interaction, *M* acquired information about $w_{1}$ and then stored it until $t_{0}$.

Broadly put, our taxonomy categorizes memory systems according to the kind of information they rely on. *Type-1* memory systems involve information only concerning the current state of the memory system, $m_{0}$. *Type-2* memory systems also involve information concerning the state of $m_{0}$, but are only guaranteed to work when some additional conditions concerning $w_{0}$ are also met. As described below, in the real world, Type-2 memory systems are time-symmetric (like in digital computers).

Finally, *Type-3* memory systems involve information based on information concerning both $m_{0}$ and $m_{1}$. (As described below, that information concerning $m_{1}$ actually follows from information concerning $m_{0}$). They are a special case of a Type-1 memory system. In fact, they are the only examples of Type-1 memory systems we know of that in the real world can accurately provide a lot of information about $w_{1}$, which is why we assign them their own type. (Below, we do not discuss any examples of Type-1 memory systems other than those that are actually Type-3.) As described below, in the real world, Type-3 memory systems are time-asymmetric (like footprints on a beach). These types of memory systems seem to capture many of the instances of memory considered in the literature, sometimes under the name of “records”. In particular, all instances of memory we know of that involve the second law of thermodynamics are Type-3 memory systems.

These three types of memory systems are closely related to three types of memory considered in [18]. Before we formally define them, in the next subsection, we present some intuitive examples of Type-2 and Type-3 memory systems to compare time-symmetric memory systems with time-asymmetric ones.

### 3.2. How Memory Systems Work

An example of a Type-2 memory system is memory in a computer. To keep our discussion independent of specific hardware implementations, we focus on abstract memory in abstract computers. We let *M* be the contents of a specific piece of Random Access Memory (RAM) that is used in a program of such a computer. The rest of the abstract computer is *W*. In particular, *W* includes the rest of the computer’s RAM outside of *M*, and the program it is running (which for argument’s sake we can imagine is implemented in a special “code segment” part of the RAM, distinct from *W*). In such a setup, *only* observing the value of $m_{0}$ does not offer us any information about $w_{1}$, i.e., the state of the rest of the computer at time $t_{1}$. The reason why a piece of RAM can nevertheless serve as a memory is that the entire system M×W consisting of the memory and the rest of the computer evolves deterministically in time. This means that we can infer something about the value of $w_{1}$ from an observation of $m_{0}$, if we also assume (or know, via prior knowledge) a salient feature of $w_{0}$. Specifically, if we know that a particular program is running on the computer at $t_{0}$ (which is information concerning $w_{0}$), then the current value of RAM, $m_{0}$, can tell us the contents of some of the rest of the computer’s RAM at $t_{1}\neq t_{0}$ (which is information concerning $w_{1}$).

Typically, we think of such computer memory as providing information about the computer’s past states. However, it is possible to evolve the system M×W forward in time as well as backwards, which means that Type-2 memory can be of the future as well as the past. (After all, knowing the program that is running and the current value of the precise part of the RAM in *m* can tell us something about the future value of some other part of the RAM, depending on the precise program.)

Notice as well that our observation of the current state of the memory, $m_{0}$, can vary arbitrarily—varying that state varies what we infer concerning $w_{1}$, and every value of $m_{0}$ provides such an inference. On the other hand, we do not consider effects on $w_{1}$ of varying the state of the world external to the memory at time $t_{0}$, e.g., of varying the program running on the computer. Instead, our inference concerning the effects of varying $m_{0}$ is preconditioned on $w_{0}$ containing a particular program, i.e., on $w_{0}$ falling within a particular subset of *W* (see pp. 749–762 of [18] for a much more detailed discussion of this kind of memory system).

If *W* is large and not fully observable, as is typical in real-life situations, then it is often impossible to determine the precise value $w_{1}$ by deterministic evolution of M×W. This might suggest that outside of the special case or digital computers, Type-2 memory systems are neither very common nor very useful. However, it is compatible with our understanding of Type-2 memory systems that the inference about $w_{1}$ is stochastic and based on a partial observation of $w_{0}$—just like with Type-1 and Type-3 memory systems (see our formal definition below for the details). If one considers these kinds of cases as well, it becomes plausible that Type-2 memory systems are a very common source of knowledge of the future. For instance, predictions of the climate on Earth based on current observations fall into this category.

Examples of Type-3 memory are footprints on a beach, impact craters, photographic film, etc. We consider the case of photographic film. Before exposure, photographic film is in a predetermined stable state, which we call its “initialized state”. Since this state can be distinguished from any state that the film can be in after exposure, we can infer from the latter, exposed state that the film interacted in a particular way with the external world. The exposed film can thus provide us with detailed information about a past state of *W*. Since the film’s state remains stable after exposure, this state of *W* can lie quite far in the past.

Knowledge from a (non-digital) photograph thus relies on an inference from both the present exposed state of the film, $m_{0}$, and its initialized state, $m_{1}$. This explains why photographic films are Type-3 memory systems. Since $m_{1}$ cannot be directly observed at time $t_{0}$, the question arises of how we can come to have knowledge of it. Below, we argue that this knowledge has to be based on the occurrence of a process that takes *M* to a known state. Crucially, as we argue, this process of initialization must increase global entropy, which implies that $m_{1}$ is a past state. Since our argument applies to all Type-3 memory systems, this means that systems of this type can only provide information about the past.

In what follows, we develop formal definitions of the three types of memory systems just sketched, and investigate them in more detail. Our definitions of Type-1, Type-2, and Type-3 memory systems provide formal elaborations of Wolpert’s [18] “b-type”, “c-type”, and “p-type” systems, respectively.

## 4. Formal Definitions of Memory Systems

As described above, we have six jointly distributed random variables indexed by time, $W_{0},W_{1},W_{2},m_{0},m_{1},m_{2}$, where the three associated times are index-ordered, i.e., either $t_{0}<t_{1}<t_{2}$ or $t_{0}>t_{1}>t_{2}$. (We do not actually make use of $W_{2}$ in what follows, except for providing some intuition.) We are interested in forming a statistical inference about $w_{1}$ based on value $m_{0}$, perhaps in combination with a constraint on the possible value of $w_{0}$. We require that the inference we draw varies depending on that value of $m_{0}$. Intuitively, whenever this is the case, we can conclude from the observed value of $m_{0}$ (perhaps in conjunction with an assumed constraint on $w_{0}$) that *M* and *W* interacted sometime between $t_{0}$ and $t_{1}$, with the interaction transferring some information about state $w_{1}$ to the memory system, *M*, where it resides until time $t_{0}$.

We can formalize the foregoing with what we call **memory systems**. We consider three types of memory systems, which differ from one another depending on whether the memory is based on value $m_{0}$, on value $w_{0}$, or on value $m_{0}$ combined with some knowledge about how the laws of physics arise in the joint dynamics of M×W.

In the rest of this paper, for simplicity, we consider the case where all state spaces are countable, e.g., due to coarse-graining. This allows for us to cast the proofs in terms of sums, using Kronecker delta functions (see also the discussion above concerning the problematic nature of assuming infinite precision information). The extension to classical uncountable spaces is straightforward. (Loosely speaking, for Euclidean spaces, the sums in our proofs become Lebesgue integrals and the delta functions become Dirac deltas. For more general kinds of classical spaces, the sums become measure-theoretic integrals, and the delta functions need to be modified accordingly. The case of quantum mechanical spaces requires more care.) In addition, overloading notation, we write the indicator function as δ(.). So for any event *A* in the implicit underlying probability measure space, δ(A) equals 1/0 depending on whether *A* is true/false.

In Section 4.1, we begin by introducing a variant of some standard information-theoretic definitions. These play a central role in our fully formal definitions of those three types of memory systems, which we present in Section 4.2.

### 4.1. Restricted Mutual Information

In general, whether state $m_{0}$ provides a memory about state $w_{1}$ depends on certain conditions concerning the joint value of $\left(m_{0},w_{0}\right)$ being met. Accordingly, our definitions involve statements of the form “If condition C concerning $\left(m_{0},w_{0}\right)$ is met, then the following mutual information will be high”. We do not model how the user of the memory system does (or does not) come to know whether condition C is met. Often, it is background knowledge, over and beyond the background knowledge that determines joint distribution $P(m_{0},w_{0},m_{1},w_{1},m_{2},w_{2})$.

To illustrate this, we consider again the example of a computer memory described above. In that example, *M* is (the state of) part of a computer’s RAM, and *W* is (the state of) the rest of the computer, including, in particular, the rest of the RAM, and so the program that is running on the computer. P(.) depends on the dynamics of the entire computer, as usual. In this example, condition C is the knowledge that some specific program is currently executing in *W*, the rest of the computer outside of the part of the RAM constituting *M*. It is the precise form of that program which, combined with the current state of the part of the RAM constituting *M*, provides information concerning the state of the rest of the RAM at some other time. We note that in this example the constraint does not specify $w_{0}$ *in toto*; many degrees of freedom of the computer are free to vary.

Intuitively, knowledge that C holds is a second, different kind of “observation”, in addition to the observation of the precise current state of *M*, the computer memory in question. The difference between the two types of observation is that we are considering the effect on what we can infer about $w_{1}$ by varying over the states $m_{0}$, while we do not consider varying over whether C holds. Again, returning to the example of a computer, we distinguish the observation of the part of the RAM that comprises *M* from the “observation” of what program is running on the rest of the computer. We are interested in how varying the former leads to different conclusions concerning the state of the external RAM at some other time. In contrast, we are not concerned with the effects of varying the program.

To formalize this distinction, for any jointly distributed pair of random variables A,B taking values a,b, respectively, we let C be some set of joint values (a,b). We define *C* to be the indicator function specifying whether (a,b)∈C. So *C* is a 0/1-valued random variable, jointly distributed with our other random variables. We indicate the joint distribution as P(a,b,c), where *c* is the value of *C*. Then, we can define the random variable,

(1) $$\begin{array}{c} I_{c}\left(A;B\right):=-\sum_{a,b}P\left(a,b|c\right)\left[\ln P(a|c)-\ln P(a|b,c)\right] \end{array}$$

Intuitively, $I_{c}\left(A;B\right)$ is the value of the mutual information between *A* and *B*, evaluated only over those (a,b) pairs where condition C does/does not hold, as specified by the value of *c*. We note that $I_{c}\left(A;B\right)$ is not the same as the mutual information between *A* and *B* conditioned on *c*,

(2) $$\begin{array}{c} I\left(A;B|C\right)=-\sum_{a,b,c}P\left(c\right)P\left(a,b|c\right)\left[\ln P(a|c)-\ln P(a|b,c)\right] \end{array}$$

Indeed, I(A;B|C) is the expectation under P(c) of $I_{c}\left(A;B\right)$.

We can illustrate this definition by returning to the example where *M* is a part of the RAM in a digital computer, while the program running in the computer is stored in some other part of the RAM which is (part of) *W*. In this example, c=1 if the joint state of the RAM *W* and the program stored in the rest of the RAM fulfills some special condition (see discussion below).

We refer to $I_{c}\left(A;B\right)$ for c=1 as the (C-)**restricted** mutual information between *A* and *B*. We write it as $I_{C}\left(A;B\right)$, with value c=1 being implicit.

Memory systems are defined in terms of *sufficient* conditions for information concerning the external world at one time to be conveyed to the memory system at another time, and we make no claims about *necessary and jointly sufficient* conditions. For this reason, in this paper, we are interested in restricted mutual information rather than conditional mutual information, with C=1 for different choices of C being sufficient conditions.

As an aside, we note that we can define variants of entropy and conditional entropy that are analogous to $I_{c}\left(A;B\right)$:

(3) $$\begin{array}{cc} H_{c}\left(A\right) & :=-\sum_{a}P\left(a|c\right)\ln P\left(a|c\right) \end{array}$$

(4) $$\begin{array}{cc} H_{c}\left(A|B\right) & :=-\sum_{a.b}P\left(a,b|c\right)\ln P\left(a|b,c\right) \end{array}$$

where, as before, c∈C is a 0-1 valued random variable specifying whether condition C holds. For any such random variable *C* and either value *c* of that random variable,

(5) $$\begin{array}{c} I_{c}\left(A;B\right)=H_{c}\left(A\right)-H_{c}\left(A|B\right) \end{array}$$

Paralleling our convention for restricted mutual information, we sometimes write the two types of restricted entropy evaluated for c=1 as $H_{C}\left(A\right)$ and $H_{C}\left(A|B\right)$, respectively. So, in particular,

(6) $$\begin{array}{c} I_{C}\left(A;B\right)=H_{C}\left(A\right)-H_{C}\left(A|B\right) \end{array}$$

in direct analogy to the relation among (non-restricted) entropy, conditional entropy, and mutual information.

As a point of notation, we often write something like “a∈C” inside a probability distribution as shorthand for the event that the value of the associated random variable C=1. Similarly, we write $I_{a\in C}\left(\ldots \right)$ as shorthand for C-restricted mutual information where variable *a* lies in set C. Furthermore, we let d∈D be some random variable. Rather than write “for all (a,b)∈C,P(d|a,b) obeys *…*”, it is convenient to write “$P_{(a,b)\in C}\left(d\;|\;a,b\right)$ obeys *…*”.

### 4.2. The Three Types of Memory Systems

**Definition** **1.** A **Type-1** memory is any stochastic process over space M×W where there is some set $M^{*}$ such that $I_{m_{0}\in M^{*}}\left(W_{1};M_{0}\right)$ is large.

**Definition** **2.** A **Type-2** memory is any stochastic process over space M×W where there is some set $W^{*}$ such that $I_{w_{0}\in W^{*}}\left(W_{1};M_{0}\right)$ is large.

**Definition** **3.** *A **Type-3** memory is any stochastic process over space M×W where:*

*(1)*  There is an $m^{†}\in M$ and a set $M^{*}$ such that $I_{m_{1}=m^{†},m_{0}\in M^{*}}\left(W_{1};M_{0}\right)$ is large.
*(2)*  There is a set $M^{\prime }\subseteq M$ such that for all $m_{0}\in M^{*}$,
  *(a)*  *$P(m_{2}\in M^{\prime }\;|\;m_{0})$ is close to 1.*
  *(b)*  *$P(m_{1}\;|\;m_{2},m_{0})$ is a highly peaked distribution about $m_{1}=m^{†}$, for all $m_{2}\in M^{\prime }$.*
  *(c)*  *$w_{1}$ is conditionally independent from $m_{2}$, given $m_{0}$ and given that $m_{1}=m^{†}$. In other words,*
$$\begin{array}{c} P\left(w_{1}\;|\;m_{0},m_{1}=m^{†},m_{2}\right)=P\left(w_{1}\;|\;m_{0},m_{1}=m^{†}\right) \end{array}$$

Item 1 of the definition of Type-3 memory systems says that if we believe for some reason that the memory is in initialized state $m^{†}$ at $t_{1}$, and if $m_{0}\in M^{*}$, then knowing precise value $m_{0}$ provides a lot of information about $w_{1}$. Intuitively, knowing both that the system was in $m^{†}$ at $t_{1}$ and that $m_{0}\in M^{*}$, we can conclude that *W* must have interacted with *M* between $t_{1}$ and $t_{0}$, with the precise relationship between $m^{†}$ and $m_{0}$ providing information about the state of *W* before that interaction started, at $t_{1}$. Item 1 says that we have reason to believe that $m_{1}$ does in fact equal $m^{†}$, and so we can use $m_{0}$ to make an inference about $m_{1}$ this way.

As established in Lemma 1 below, Lists 2a and 2b of Definition 3 then provide a set of properties of the joint probability distribution that justify that belief concerning $m_{1}$, the state of the memory at $t_{1}$, given only the fact that the present state of the memory system is in $M^{*}$. (Item 2c is a simplifying assumption, made for expository convenience).

Theorem 1 below then uses Lemma 1 to show that when the conditions for a Type-3 memory system hold, $I_{m_{0}\in M^{*}}\left(W_{1};M_{0}\right)$ is large. So only knowing something about the *current*, $t_{0}$ value of *m* is sufficient to conclude that it is statistically correlated with the value of *w* at the *different* time, $t_{1}$. This proves that Type-3 memory systems are a special case of Type-1 memory systems. In fact, as also discussed below, Type-3 memory systems are an especially important special case of a Type-1 memory system, since they can be considered as a formalization of the primary type of memory system that is considered to be a “record of the past” in the previous literature on the epistemic arrow of time. The second law of thermodynamics seems to play a crucial role in allowing the properties defining Type-3 memory systems (in particular, Item 2b) to occur in the real world. In contrast, the second law does not arise at all in Type-2 memory systems.

**Lemma** **1.** *For a Type-3 memory,*

*(1)*  For any $m_{0}\in M^{*}$ and any $w_{1}$,
(7) $$\begin{array}{c} P\left(w_{1}\;|\;m_{0}\right)≃P\left(w_{1}\;|\;m_{0},m_{1}=m^{†}\right) \end{array}$$
and since this holds for all $m_{0}\in M^{*}$,
(8) $$\begin{array}{cc} P(w_{1}\;|\;m_{0},m_{0}\in M^{*}) & ≃P(w_{1}\;|\;m_{0},m_{1}=m^{†},m_{0}\in M^{*}) \end{array}$$
*(2)*  *For any $m_{1}$,*
(9) $$\begin{array}{cc} P(m_{1}|m_{0}\in M^{*}) & ≃\delta (m_{1},m^{†}) \end{array}$$
*(3)*  *For any $m_{0}$,*
(10) $$\begin{array}{cc} P(m_{0}|m_{0}\in M^{*}) & ≃P(m_{0}|m_{1}=m^{†},m_{0}\in M^{*}) \end{array}$$
*(4)*  *For any $w_{1}$,*
(11) $$\begin{array}{cc} P(w_{1}\;|\;m_{0}\in M^{*}) & ≃P(w_{1}\;|\;m_{1}=m^{†},m_{0}\in M^{*}) \end{array}$$

**Proof.**  For any $m_{0}\in M^{*}$ in a Type-3 memory, we can expand

(12)P(w1|m0)=∑m1,m2P(m2|m0)P(m1|m2,m0)P(w1|m0,m1,m2)(13)≃∑m1,m2P(m2|m0)δ(m2∈M′)∑m^2∈MP(m^2|m0)δ(m^2∈M′)P(m1|m2,m0)P(w1|m0,m1,m2)(14)=∑m1,m2P(m2|m0)∑m^2∈MP(m^2|m0)δ(m^2∈M′)δ(m2∈M′)P(m1|m2,m0)P(w1|m0,m1,m2)(15)≃∑m1,m2P(m2|m0)∑m^2∈M′P(m^2|m0)δ(m^2∈M′)δ(m2∈M′)δ(m1,m†)P(w1|m0,m1,m2)(16)=∑m2P(m2|m0)P(w1|m0,m1=m†,m2)(17)=∑m2P(m2|m0)P(w1|m0,m1=m†)(18)=P(w1|m0,m1=m†)

where the second line expands the first conditional distribution in the summand and uses Item 2a of the definition of Type-3 memory systems, the fourth line uses Item 2b, the fifth line collapses the conditional distribution that was expanded in the second line, and then the sixth line uses Item 2c. This establishes Lemma 1(1).

Next, we expand

(19)P(m1|m0∈M*)=∑m2P(m1|m0∈M*,m2)P(m2|m0∈M*)(20)≃∑m2P(m1|m0∈M*,m2)P(m2|m0∈M*)δ(m2∈M′)(21)≃∑m2δ(m1,m†)P(m2|m0∈M*)δ(m2∈M′)(22)=δ(m1,m†)

where the second line uses Item 2a of the definition of Type-3 memory systems, and the third line uses Item 2c. This establishes Lemma 1(2).

Next, we use Lemma 1(2) to expand

(23)P(m0|m0∈M*)=∑m1P(m0|m0∈M*,m1)P(m1|m0∈M*)(24)≃∑m1P(m0|m0∈M*,m1)δ(m1,m†)(25)=P(m0|m0∈M*,m1=m†)

This establishes Lemma 1(3).

Finally, we apply $\sum_{m_{0}}P\left(m_{0}|m_{0}\in M^{*}\right)$ to both sides of Equation (8), and then use Equation (10) to replace $P(m_{0}|m_{0}\in M^{*})$ in the right-hand sum. This establishes Lemma 1(4). □

We can use Lemma 1 to derive the following result, and thereby prove that systems obeying the four properties of Type-3 memory systems are in fact a special case of Type-1 memory systems, as claimed above.

**Theorem** **1.** $I_{m_{0}\in M^{*}}\left(W_{1};M_{0}\right)$ is large in any Type-3 memory system.

**Proof.**  Using Lemma 1(1) twice allows us expansion

(26) $$\begin{array}{cc} I_{m_{0}\in M^{*}}\left(W_{1};M_{0}\right) & =\;-\;\;\sum_{m_{0},w_{1}}P\left(m_{0},w_{1}|m_{0}\in M^{*}\right)\left[\ln P\left(w_{1}|m_{0}\in M^{*}\right)-\ln P\left(w_{1}|m_{0},m_{0}\in M^{*}\right)\right] \end{array}$$

(27) $$\begin{array}{cc} & ≃\;-\;\;\sum_{m_{0},w_{1}}P\left(m_{0},w_{1}|m_{0}\in M^{*}\right)\left[\ln P\left(w_{1}|m_{0}\in M^{*}\right)-\ln P\left(w_{1}|m_{0},m_{1}=m^{†},m_{0}\in M^{*}\right)\right]\\ & ≃\;-\;\;\sum_{m_{0},w_{1}}P\left(m_{0}|m_{0}\in M^{*}\right)P\left(w_{1}|m_{0},m_{1}=m^{†},m_{0}\in M^{*}\right) \end{array}$$

(28) $$\begin{array}{cc} & \;\;\;\times \left[\ln P\left(w_{1}|m_{0}\in M^{*}\right)-\ln P\left(w_{1}|m_{0},m_{1}=m^{†},m_{0}\in M^{*}\right)\right] \end{array}$$

Next, we can use Lemma 1(3) and then Lemma 1(4) to approximate Equation (28) as

Im0∈M*(W1;m0)≃∑m0,w1P(m0|m1=m†,m0∈M*)P(w1|m0,m1=m†,m0∈M*)(29)×lnP(w1|m0∈M*)−lnP(w1|m0,m1=m†,m0∈M*)≃∑m0,w1P(m0|m0∈M*,m1=m†)P(w1|m0,m1=m†,m0∈M*)(30)×lnP(w1|m1=m†,m0∈M*)−lnP(w1|m0,m1=m†,m0∈M*)=∑m0,w1P(m0,w1|m1=m†,m0∈M*)(31)×lnP(w1|m1=m†,m0∈M*)−lnP(w1|m0,m1=m†,m0∈M*)(32)=Im1=m†,m0∈M*(W1;M0)

Finally, plugging in 1 of the definition of memory systems, we conclude that $I_{m_{0}\in M^{*}}\left(W_{1};m_{0}\right)$ is large. □

Theorem 1 establishes that in a Type-3 memory system, so long as $m_{0}\in M^{*}$, the precise state, $m_{0}$, is informative about state $w_{1}$. So whenever that condition is met, the current state of memory system *M* is a *memory* of $w_{1}$, the state of the external world at $t_{1}$, in the sense described in preceding sections.

### 4.3. Illustrations of Our Formal Definitions

In this subsection, we illustrate real-world examples of Type-2 and Type-3 memory systems to compare the formal definitions of time-symmetric and time-asymmetric memory systems.

We can illustrate the definition of Type-2 memory systems using the above example of computer memory. We recall that in that example, *M* is one part of the RAM of the computer, while *W* is the rest of the RAM, including, in particular, the part of the RAM that contains the program currently running on the computer. More precisely, we write the state space of the computer as $Z=(M,Z^{2},Z^{3})$, where $z^{2}\in Z^{2}$ specifies the particular program currently running (i.e., a particular interval of the coding segment of the computer), and m∈M is a separate part of the RAM, offering the value of one of the variables potentially modified by the running of that program. $Z^{3}$ is then the rest of the RAM and other variables in the computer whose value is not involved in specifying the program.

So in this computer memory example, *W* is $\left(Z^{2},Z^{3}\right)$. However, it is convenient to parameterize elements of *W* by their value of $Z^{2}$, coarse-graining over possible values of $Z^{3}$. In particular, $W^{*}$ is all states of $\left(Z^{2},Z^{3}\right)$ where $z^{2}$ contains particular program $z^{2}$, a program that allows inference from the current state of the memory, $m_{0}$, about the past and/or future of variable *m*. This is particularly clear in cases where the typical values of $z^{3}$ have no effect on the dynamics of $\left(m,z^{2}\right)$, while the joint values of $\left(m,z^{2}\right)$ *can* affect the dynamics of $z^{3}$. Concretely, in such a case, the state of the RAM *m* is specified in some time outside of interval $\left[t_{0},t_{1}\right]$, and during that interval it can affect the value of some other part of the RAM, $z^{3}$, but the value of $z^{3}$ cannot affect the value of *m* during that interval. So knowing $m_{0}$ and $z_{0}^{2}$, the current value of $z^{2}$, suffices to draw inferences about $z_{1}^{3}$, the state of $z^{3}$ at time $t_{1}$.

More generally, in many Type-2 memory systems, M×W is a semi-closed system, not able to be affected by the state of the rest of the physical universe during interval $\left[t_{0},t_{1}\right]$. In such a case, since the laws of physics are deterministic and invertible in any semi-closed system, the joint dynamics of M×W is deterministic during $\left[t_{0},t_{1}\right]$. Type-2 memory systems with this feature can result in almost perfect memory, as described in Section 3.2. It is important to note, though, that we do *not* require that there be a decomposition of *W* into two such variables $z^{2},z^{3}$; we assume that decomposition here for illustrative purposes only.

It might be helpful to illustrate these points using the example of the joint state of the planets in the solar system. First, we note that the planets are not a memory at all of their own future state; that would mean using their state now to derive information about their own state at a different time, whereas we generally assume that the memory and the system that it “remembers” are different from each other. One might suppose instead though that the behavior of some of the planets can provide some information about the others at both past and future times.

We note, though, that memory systems are systems whose state *at a single moment of time*, $t_{0}$, provides information about some other system at a different time. So for the supposition to hold, we need to interpret “behavior” to mean some characteristic of some of the planets at a single moment. However, the phase space positions of the planets at a single moment do not provide such a characteristic; we need to also know the acceleration vectors of those planets, not just their positions and velocities. If those acceleration vectors were included in the state space of *M*, then (and only then) *M* could serve as a Type-2 memory system of the future state of the other planets (see also Point 3 in the discussion of Type-3 memory systems below).

We can illustrate the different parts of the definition of Type-3 memory systems with the example of a line of footprints across a beach. In this example, *M* is the set of all versions of the *pattern* on the surface of the beach—smoothed, with a single line of footprints, churned by many people walking across it, etc. $M^{\prime }$ is all versions of the patterns on the surface of the beach that are not in some unusual state that would prevent the beach from being swept smooth. In particular, $M^{\prime }$ does not contain any versions of the (pattern on the surface of a) beach that are so badly churned that it would not be possible for them to be swept smooth by ocean waves during a high tide. (So, in particular, patterns in which there is huge hole, many tens of meters deep, do not lie in $M^{\prime }$.) $M^{*}$ is the set of all versions of the beach that are completely smooth, having been swept by ocean waves, during a high tide—with the possible (!) exception that there is some very clearly defined line of footprints across the surface of the beach. Finally, $m^{†}$, the “initialized state”, is the beach right after it has been smoothed by ocean waves. (N.b., strictly speaking, $m^{†}$ is not a single state, but a set of very similar states. To simplify the exposition, we often treat a set of very similar states as though they were a single state, as was also performed in the example above of a computer memory.) In contrast, *W* is the set of all other systems on the surface of the Earth that could conceivably interact with the surface of the beach some time in the interval between $t_{0}$ and $t_{2}$.

Item 1 reflects the fact that if we know both that the beach surface was smooth at $t_{1}$ and that it currently is smooth except for a single line of footprints, then we can conclude that a person must have walked across the beach some time between $t_{1}$ and $t_{0}$, with the precise pattern of those footprints providing information about that walk.

Item 2a of the definition of Type-3 memory systems then tells us that so long as the current pattern on the beach is a single line of footprints, we have no reason to suppose that the surface of the beach was in some unusual state that could not be wiped smooth just before the most recent high tide.

Item 2b of the definition of Type-3 memory is enforced by the second law of thermodynamics. More precisely, the collapsing of the state space of *M* described in Item 2b involves coupling *M* with some third system, *K*. The second law drives an irreversible process that increases total entropy in M×K while at the same time collapsing *M* from subset $m_{2}\in M^{\prime }$ down to the precise value of $m_{1}=m^{†}$. (This is related to what was called “external initialization” in [18]).

Concretely, a beach is initialized as $m^{†}$ when it is smoothed by the ocean waves driven by the tide. *K* is those ocean waves, lapping the beach during this re-initialization of the state of the beach. Projected down to the states of the beach, that smoothing of the beach by ocean waves is a non-invertible process, driven by the second law. This reliance on the second law, of course, is precisely why this example of a Type-3 memory system is time-asymmetric (as noted above, Item 2c is assumed simply for expository convenience, and clearly holds for this example of a beach).

We note that just like with Type-2 memory systems, with Type-3 memory systems there is an implicit assumption that *W* is a minuscule portion of the full physical universe (more precisely, we assume that the probability that variables in the physical universe that lie outside of *W* are in a state that would cause them to interfere with our inference is effectively zero). Furthermore, it is implicitly assumed that the dynamics of those degrees of freedom of *W* we are concerned with are effectively isolated from that rest of the universe (aside from the possible interaction with system *K*). This can be formalized in terms of a prior distribution over the state of the full universe, including *W* as a subsystem. For example, this assumption implies that the prior probability that the sand on the beach was manipulated by powerful aliens to make it appear as though people had walked over a smooth beach is small.

We note also that the fact that the distribution over *m* at $t_{1}$, the end of the initialization process, is (almost) a delta function about $m^{†}$ means that the distribution over *M* at that time, when it is in its initialized state, has *low entropy*. It is the distribution over the joint state, M×K, whose entropy increases in the initialization of *M*.

This is a crucial point. When the beach has been swept smooth, the precise three-dimensional configuration of all the sand grains inside of a beach is close to thermal equilibrium (for the Hamiltonian function given by the gravitational field of the Earth). That does not change the fact that the pattern on the surface of a smooth beach has a very *low* entropy, when considered as a distribution over the space of all possible patterns on the surface of the beach. The inference underlying memory systems—Theorem 1 above—concerns that space of all possible patterns on the surface on the beach. It does *not* concern the thermodynamic entropy of the underlying three-dimensional configuration in the Earth’s gravitational field.

A flash drive is another example of Type-3 memory that provides an even more graphic illustration of how the initialized, ready state of *M* can have low entropy. Here, $M=(Z_{1},Z_{2})$, where $Z_{1}$ is the contents of the flash drive’s binary memory, and $Z_{2}$ is other attributes of the physical flash drive, in particular whether it has physical damage (e.g., puncture holes in the flash drive’s casing). $M^{*}=M^{\prime }$ is all joint states in $\left(Z_{1},Z_{2}\right)$ where ($Z_{2}$ has a value indicating that) the flash drive is undamaged. $m^{†}$ is the “wiped clean”, all-zeros joint state of the flash drive’s entire memory, i.e., of $Z_{1}$.

The important thing to note is that this wiped-clean state where the bits are all zeros with probability one is *minimal* entropy. It is produced by coupling the flash drive with an external, electronic initializing system, *K*, in a “wiping clean” process of the contents of the flash drive. That initialization process relies on the second law of thermodynamics to increase the joint entropy of the flash drive *and the electronic initializing system*. So just like the beach was wiped smooth by the action of waves during a high tide, which increased the joint entropy of the waves and the beach while reducing the marginal entropy of just the beach, the flash drive was wiped clean by action of the electronic initializing system, which increased the joint entropy of the initializing system and the flash drive’s bits while reducing the marginal entropy of just the flash drive’s bits.

As an alternative, we could reformulate these examples of Type-3 memory systems not to involve an external system, *K*. We can do this by “folding *K* in” to the definition of *M*. In the example of a beach surface memory system, this means redefining *M* to be the joint state of pattern on the surface of the beach *and the precise physical state of the ocean lapping that beach*.

We end by noting that it is straightforward to formalize many other examples of memory systems considered in the literature (in particular, those considered in [18]) as Type-3 memory systems. For pedagogical reasons, we sketch some of them here, omitting detailed discussion. We note that while it would in principle be possible to provide a precise quantitative characterization of these and other systems, it may not be easy to do so in practice.

1. Consider an image on a chemical photographic film in an instant camera. *M* is the possible patterns on the surface of the film; $M^{*}$ is all such patterns aside from those that indicate the camera holding the film was incorrectly exposed to the outside world, e.g., resulting in a fogged image on the surface of the film. $m^{†}$ is the initialized state of the film, with no image, before exposure of any sort. It has low entropy, and is formed in an entropy-increasing chemical initialization process that involves some external set of chemicals, *K*. *W* is an external photon field, which results in an image being made some time between $t_{1}$ and $t_{0}$ if the camera exposes the film correctly, i.e., if $m_{0}\in M^{*}$.
2. Suppose we come across a cave and find that inside of it, some of the stones scattered about the floor (which evidently had originally been part of the roof) are arranged in letters, spelling “Help!”. In this case, *M* is (a coarse-graining of) the possible patterns of stones on the floor of the cave. $m^{†}$ is the pattern where the stones are scattered uniformly randomly. We rely on the second law to presume that the joint state of the cave (including, in particular, its roof and the pattern of stones on its floor) was in $m^{†}$ some time in the past. This allows inferring that some subsystem of *W* (in this case, some English-speaking human) interfered with *M* at some time between when *in the past* it was initialized to $m^{†}$, and the present, when the stones spell out “Help!”. Intuitively, this example is just like footprints on the beach, where the analog of the smoothed beach surface is the initially random positions of stones on the cave floor (notice that this is a high-entropy state!), and the analog of the trail of footprints is some of the stones being arranged to spell “Help!”.
3. Suppose we took some photographs through a telescope of the positions of the planets of the solar system which (together with other recorded information gathered from different positions on the surface of the Earth) allow us inferring their current positions and velocities. Those photographs and recordings are jointly a Type-3 memory system (see discussion just above of the Type-3 memory system of an image on a photographic film). Note that we can evolve what we infer from the current state of this memory system—the current phase space position of the planets in the solar system—into *the future*, after time $t_{0}$. In this, the current value, $m_{0}$, of the memory system provides information about the future, not just the past. However, the key is that the recordings are a Type-3 memory system, and they provide information about the (recent) past. The fact that that information provides predictions concerning the future is a red herring.

### 4.4. Discussion of Our Formal Definitions

In this subsection, we briefly discuss some aspects of the formal definitions of the various types of memory systems.

First, we note that while there is no need to do so here, we could replace phrases like “$I_{m_{0}\in M^{*}}\left(W_{1};M_{0}\right)$ is large” with more formal expressions. For example, we suppose that both $|M^{*}|$ and |W|, the number of states in $M^{*}$ and in *W*, respectively, are finite. Then, we could replace that phrase by saying that $I_{m_{0}\in M^{*}}\left(W_{1};M_{0}\right)$ is close to $\min(\ln|M^{*}|,\ln|W|)$, its maximum possible value.

We note also that in Type-1 and Type-3 memory systems, we allow the possibility that we can know the value of $m_{0}$ even if it is outside of $M^{*}$. We even allow for the possibility that there would be nonzero mutual information between the value of $m_{0}$ and that of $w_{1}$ for $m_{0}\notin M^{*}$. However, our analysis concerns what happens when $m_{0}\in M^{*}$. (Mutatis mutandis for values of $w_{0}$ outside of $W^{*}$ in the case of Type-2 memory systems.)

In real-world Type-3 memory systems, often, *m* does not change in $\left[t_{2},t_{0}\right]$ except at the time of its interaction with *W*. While we do not require this, it has the practical advantage that it simplifies the calculation by the memory’s user of the relationship between the value of $w_{1}$ and $m_{0}$. It also means that we do not need to be precise about when times $t_{1}$ and $t_{2}$ are.

It is important to realize that system *K* in Type-3 memory systems, which couples with *M* in an entropy-increasing process to send $M^{\prime }$ to $m^{†}$, does not explicitly occur in the definition of Type-3 memory systems. Rather, it arises *in practice*, as part of the underlying process that enforces the requirement in 2a that conditional distribution $P(m_{1}\;|\;m_{2},m_{0})$ is peaked about $m_{1}=m^{†}$. In turn, that requirement is only relevant under the supposition that $m_{0}\in M^{*}$ and $m_{2}\in M^{\prime }$.

There are many important ways that the analysis in this paper extends beyond/modifies the analysis in [18], which was written before the revolutionary advances of the last two decades of stochastic thermodynamics. Like all considerations of the thermodynamics of computation at the time, it was based on semi-formal reasoning, grounded in equilibrium statistical physics. However, computers are actually very far from thermal equilibrium, with the result that the understanding of the relationship between logical and thermodynamic irreversibility at the end of the twentieth century and its implications for the thermodynamics of computation was mistaken. Our paper does not rely on that mistaken earlier understanding, and is fully consistent with our modern understanding of statistical physics (see [31,32] and references therein for an introduction to the modern understanding of the relationship between logical and thermodynamic irreversibility).

Another important feature of [18] is its repeated invocation of the Maxent principle of Jaynesian inference. In this paper, we do not use Maxent. Indeed, we are careful to make no arguments about how it is that the user of a memory system may arrive at the probability distributions they are using. In particular, it is worth noting that in this paper, we make no a priori assumption that $P(m_{0},m_{1},w_{0},w_{1})$ has full support (fn. 9, see [18]).

## 5. Memory Systems, Records, and the Epistemic Arrow

Of the three types of memory systems we considered, Type-3 systems are the only ones that, at least in all of their instances we know of in our physical universe, are time-asymmetric, in that they can only provide information about the past. As we explained, Type-3 memory systems rely on the second law, in that they exploit the fact that an increase in global entropy reliably takes the (local) memory system to its initialized state, which is a known state at $t_{1}$.

While we did not prove it, we note that in practice, the only way the need for the second law can be circumvented without major sacrifice in the accuracy of the memory is if we have detailed knowledge of those “dynamically relevant” degrees of freedom in the present state of *W* that (perhaps together with the precise state of *M*) determine the dynamics of *M*. In practice, as in the computer example of Type-2 memory systems, we in fact have a way to (almost) deterministically calculate the joint dynamics of M×W.

We note that these requirements do not preclude the possibility that *W* is extraordinarily large. (For example, a modern cloud computer system has tens of thousands of servers, each with ∼10^15^ (?) dynamically relevant degrees of freedom. So setting *M* to be part of the memory of just one of those servers, |W| is on the order of Avogadro’s number. Yet, such computer systems are examples of Type-2 memory systems.) However, running a Type-2 memory system with a large *W* seems to require a huge number of energy barriers keeping trajectories of $M\times Z_{2}$ well separated during the evolution of the joint system, with high probability, i.e., such systems use a huge amount of error correction; this is certainly true in cloud computers. Systems with this property seem to only arise with careful engineering by humans. In contrast, memory systems like footprints on a beach do not rely on anything close to that number of energy barriers, allowing the stochastic process governing the dynamics of microstate trajectories spreading out more readily. This may be why they can occur in systems that are not artificially constructed; see discussion of the Past Hypothesis in Section 6.

In what follows, we discuss whether Type-3 memory systems might correspond to records. After this, we argue that human memory is plausibly Type-3, which means that our analysis is suitable for explaining the epistemic arrow of time.

Common examples of records, such as impact craters, footsteps on the beach, and photographic film, are Type-3. Furthermore, Albert and Loewer claim that records require a ready state, and the initialized state formalized in our definition of Type-3 memory systems as $m^{†}$ is such a ready state. Does this mean that Type-3 memory systems can be interpreted as a formalization of records? In the absence of a precise definition of records, this question is difficult to answer. We believe that for this interpretation to work, one needs to assume that it is true by definition that records rely on an initialized state—otherwise, we do not see a clear way to distinguish records from Type-2 memory systems. If this assumption is made, then our analysis (which in turn builds on the work in [18], as described above) might provide a new basis for understanding Albert and Loewer’s claim that the epistemic arrow is constituted by the temporal asymmetry of records which avoids the problematic aspects of their argument (see Section 2).

At present, the physical details of how the human brain stores information are largely unknown. This makes it difficult to determine the type of memory system the human brain represents. Nevertheless, there are reasons to think that human memory is Type-3. First, there is the simple fact that human memory only provides information about the past. Since Type-3 memory systems are the only memory systems that exhibit this kind of temporal asymmetry, this suggests that human memory is Type-3. Second, human memory in the primary sense resides in the brain—we might call this “internal memory”. But humans also remember things indirectly by means of external devices, such as photographs, books, or digital storage media—we might call this “external memory”. External memory, at least if it concerns information about events occurring outside of computers, is typically Type-3 (our discussion in Section 4.3 demonstrates this for some such systems, namely photographs and flash drives). This makes it possible for such memory to store very detailed information. Internal memory, too, often provides us with highly detailed information about specific events. An important aspect of the psychological arrow of time is that we experience the future as “open” and the past as “fixed” ((see [18], pp. 776–778) for further discussion of the relation between this aspect of the psychological arrow and the epistemic arrow). It is plausible that the fact that we have such detailed memories of the past is at least part of the cause of this apparent openness of the future and fixity of the past (see [33] for a deeply contrarian view, arguing that time does indeed flow). The fact that internal memory can provide such detailed information supports the idea that it is Type-3. If this is the case, then our analysis is suitable for explaining how the epistemic arrow arises from the second law of thermodynamics.

## 6. The Past Hypothesis and the Second Law

Another important issue arises from the discussion at the end of Section 4.4: how exactly is it that the user of the memory comes to “know” the joint distribution in the first place? Does acquiring that knowledge itself rely on memory of past observations of the physical world? This is an extremely subtle issue, which ultimately requires engaging with the formal impossibility of inductive inference [34,35,36]. *If* the joint probability distributions of M×W at multiple moments in time has the structure of a Type-3 memory system formally defined in Section 4.2, then the relevant mutual information can in principle be exploited. Moreover, sidestepping the problem of inductive inference [36], speaking purely as empirical scientists, it seems likely that natural selection has guided (the genes encoding) our biological memories to assume those distributions in order to increase our biological fitness. But in this paper, we do not grapple with these issues.

An important unresolved problem involves the asymmetry of the second law, which appears to be fundamental to (the asymmetry of Type-3 memory and therefore) the asymmetry of human memory. We are sympathetic to the idea, which is also present in Albert and Loewer’s account, of grounding the second law in the “Past Hypothesis”. However, all arguments in the literature for how that hypothesis results in the second law have been informal. When we consider the issue more formally, we find that there are some problematic aspects with these arguments.

To see this, first, we note that essentially by definition, all the data we can *directly* access when performing any kind of scientific reasoning is in the form of observations of the values of variables solely at a single moment, which we conventionally refer to as the “present”, $t_{0}$. However, similarly to all the other dynamical laws of physics, the second law concerns the value of the entropy of the universe across a *range* of times, *t*, differing from the present, $t_{0}$. In addition (and in contrast to almost all other dynamical laws of physics), the second law is stochastic. Combining these results, we see that when we are investigating the formal basis of the second law, we are implicitly analyzing conditional distribution $P(S_{t}\;|\;{data}_{t_{0}})$ where $S_{t}$ is the entropy of the universe at time *t*, and data $t_{0}$ is all of our empirical data at present.

It is actually a subtle issue (especially from a philosophical perspective) to quantify what the precise implications of our current observations are concerning $S_{t}$ for multiple times *t*. However, as a simplifying assumption/approximation, the Past Hypothesis assumes we can distill our present data to (effectively) exact values of current entropy $S(t_{0})$ and also of the entropy at the time of the Big Bang, $S(t_{BB})$. (Arguably, the value of $S_{BB}$ cannot be estimated from our current observations with as high certainty as $S_{t_{0}}$, since all of the theorizing of modern cosmology must itself be inferred from current observations in order to make the estimate. It is (far) beyond the scope of this paper to try to quantify our relative certainty in those two estimates). These two approximations transform the distribution we are interested in:

(33) $$\begin{array}{c} P\left(S_{t}\;|\;{\mathrm{data}}_{t_{0}}\right)\to P\left(S_{t}\;|\;S_{t_{0}},S_{BB}\right) \end{array}$$

The Past Hypothesis proceeds to stipulate that $S_{BB}\ll S_{t_{0}}$. The argument progressing from this point to the second law has several successive parts. First, loosely following Boltzmann’s derivation of the H theorem, the argument (implicitly) models the dynamics of the entropy of the universe as a first-order Markov process, either a Focker0–Planck or a jump process, depending on the state space under consideration [37,38]. (We note that this assumption of a Markov process ignores complications arising from quantum mechanics and general relativity. We are also ignoring the precise type of coarse-graining being used (assuming we are not using string theory or the like to perform the analysis). Nonetheless, these kinds of assumptions underlie the standard formulation of the Past Hypothesis, and so we use them here.) To be consistent with the time-symmetry of the microscopic laws of physics, this Markov process must itself be time-symmetric (this symmetry is the starting point of Loschmidt’s paradox).

Now, formally speaking, a first-order Markov process only has a single conditioning value of the random variable, not two. Yet the distribution we are interested in is conditioned on the value of random variable $S_{t}$ at two times, $t_{BB}$ and $t_{0}$. The conventional form of the argument uses informal reasoning to sidestep this issue. It tries to make the case that since $S_{BB}\ll S_{t_{0}}$, the trend of the average value of $P(S_{t}\;|\;S_{t_{0}},S_{BB})$ must be monotonically decreasing as *t* shrinks to smaller values than $t_{0}$. This is then taken to further imply that for all times $t_{1},t_{2}$ such that $t_{BB}<t_{2}<t_{1}<t_{0}$, $P(S_{t_{1}}\;|\;S_{t_{0}},S_{BB},S_{t_{2}})$ is strongly biased to values $S_{t_{1}}>S_{t_{2}}$ (implicitly, this is the form of the second law used above in the analysis of Type-3 memory systems).

Let us suppose, as in the Past Hypothesis, that based on current data we can know the values of $S_{BB}$ and $S_{t_{0}}$, that $S_{BB}\ll S_{t_{0}}$, and that the associated distribution of interest is $P(S_{t}\;|\;S_{t_{0}},S_{BB})$. What happens if we try to use fully formal reasoning at this point, investigating the form of such a distribution conditioned on two separate events when the underlying Markov process is time-symmetric?

To calculate the marginal distributions of a random variable evolving under a time-symmetric Markov process given its values at *two* times, we must use a “Brownian bridge” [37]. In general, because the underlying stochastic process is symmetric, the Brownian bridge calculation leads to the conclusion that in the very recent past, just before the present, the entropy of the universe was *not* likely to be lower than it is today, but is actually more likely to have been slightly *higher* than it is today. Then, as we look further into the past from the present, the trend line “turns over”; the expected entropy starts decreasing, and then falls precipitously, to reach the conditioning, extremely low value in the distant past, in broad accord with the Past Hypothesis.

How can this be reconciled with the second law? In mesoscopic systems, with a relatively small number of degrees of freedom, the Markov process is diffusive enough for this “turnover” effect to be readily observable. The result is that the second law of thermodynamics in fact violated if one moves a very small amount into the past towards a point in time with a known, very low value of entropy *if there are few degrees of freedom in the universe*.

In the macroscopic system of our actual, cosmological universe, though, we would expect the diffusion term in the Markov process to be much smaller than the drift term, i.e., for the variance of the dynamics to be far smaller than the trend. If there were enough degrees of freedom, there might not even by an increase in expected entropy as we move into the past from the present. The only effect of the Brownian bridge might be to elevate the entropy in the recent past higher than it would be if we did only know the entropy at the Big Bang, rather than also knowing the current entropy. Presumably, it would require extremely careful and precise experiments to discern this effect at the macroscopic scale of our universe.

These phenomena concerning time-symmetric Markov processes are illustrated in the following example. For pedagogical reasons, this example replaces entropy with another quantity that undergoes a time-symmetric Markov process:

**Example** **1.** *Suppose we have an N-dimensional grid where each of the N coordinates has 2L+1 possible values, −L,…,−1,0,1,…,L. Impose periodic boundary conditions, so the grid lies on an N-dimensional torus. Consider a discrete time simple random walker over that grid who moves in an unbiased manner. Write the position of the random walker at timestep t as $x\left(t\right):=\left(x_{1},\ldots ,x_{N}\right)\left(t\right)$ (so in the step from time t to t+1, the walker has equal probability of moving to any one of the neighbors of x(t), all of which have Hamming distance 1 to x(t)).*

*Since the dynamics follows a random walk, it is a Markov process. Moreover, that process is ergodic, so the long-term probabilities are uniform over the entire grid. Suppose that the distribution over possible locations of the random walk reached this stationary point at some point in the infinite past. Therefore, the unconditioned probability distribution of the position of the walker at any time t we are considering, i.e., marginal distributions P(x(t)), is also at that stationary point, and the marginal distribution is uniform over the entire grid at all times t.*

*Consider the set of all cubes defined by the grid that are centered on the origin. Each of those cubes has a different radius, d, and therefore a different number of grid points in its surface. So any point x with coordinates $x_{i}$ lies on the surface of the cube with radius $d=\max_{i}\left|x_{i}\right|$. The area of that surface (i.e., the number of grid points contained in that surface) is the difference between the volume of that cube and the volume of the cube lying just inside of it,*

(34)A(d)=[d]N−[d−2]N(35)=2N[d]N−1−2N(N−1)[d]N−2+…(36)=2N[d]N−11−(N−1)d+…(37)∼2N[d]N−1in the limit that d/N→∞

*Note that this is not the same as the surface area of cube in $R^{N}$ with radius d.*

*There is no “energy” in this scenario, so we cannot define the Boltzmann entropy of a microcanonical ensemble as the log of the area of a shell of fixed energy, as in conventional statistical physics. However, we can instead consider the Boltzmann entropy for shells given by the sets of points lying on the cube surfaces with successive values of d (so d plays the role that energy plays in the conventional microcanonical ensemble).*

*For this choice, the Boltzmann entropy for point x lying on an N dimensional grid is*

(38) $$\begin{array}{c} S\left(x\right)=\ln A\left[\max_{i}x_{i}\right] \end{array}$$

*Given any (stochastic) trajectory of the random walker, x(t), write d(t) for the radius of the cube whose surface contains x(t), and write the associated Boltzmann entropy as S(t). As an example, for N=2, $S\left(t\right)=\ln\left(4\max(|x_{1}\left(t\right)|,|x_{2}\left(t\right)|)\right)$.*

*Since the random walk is unbiased and (by time-translation invariance) at any time t the marginal distribution is the stationary point of that walk, it follows from symmetry that the Markov kernel is symmetric in time, i.e.,*

(39) $$\begin{array}{c} P(x(t+1)=a|x(t)=b)\;=\;P(x(t+1)=b|x(t)=a) \end{array}$$

*In fact, there is an iff, in that if Equation (39) holds, then P(x(t))=P(x(t+1)), i.e., the marginal distribution is a stationary point of the dynamics at t. (To prove this well-known result, consider any two random variables A and B, with the same space of possible values. Write $P_{A}$ and $P_{B}$ for the respective marginal distributions, and write K(b,a) for conditional distribution P(B=b|A=a) and the Bayesian inverse as $\hat{K}\left(a,b\right)=P\left(A=a|B=b\right)$. Using conventional shorthand, $P_{B}=KP_{A}$ and $P_{A}=\hat{K}P_{B}$. Combining the results, $P_{A}=\hat{K}KP_{A}$. If $K=\hat{K}$, it follows that $P_{A}$ is a stationary point of K. Note that this has nothing to do with Markovian dynamics; in this case, A=x(t) and B=x(t+1), but the argument here is more general.)*

*Given Equation (39) and the fact that the random walk is time-homogeneous, we conclude that for any value $k^{\prime }$, diameter d(t) (and therefore value S(t)=k), and positive integer q,*

(40) $$\begin{array}{c} P\left(S\left(t-q\right)=k^{\prime }\;|\;S\left(t\right)=k\right)\;=\;P\left(S\left(t+q\right)=k^{\prime }\;|\;S\left(t\right)=k\right) \end{array}$$

*This confirms that the Markov process over entropy values is indeed time-symmetric.*

*Measuring units of t in years, define $t_{BB}=-1.3\times 10^{14}$ and $t_{0}=0$. Suppose as well that the entropy, then, $S(t_{BB})$, is quite small (much smaller than the maximal value of S, ${\left(2L+1\right)}^{N}-{\left(2L-2\right)}^{N}$). Then,*

(41) $$\begin{array}{c} E_{P}\left(S\left(t_{0}\right)\;|\;S\left(t_{BB}\right)\right)>S\left(t_{BB}\right) \end{array}$$

*This is the essence of the traditional argument that the Past Hypothesis results in the second law.*

*On the other hand, suppose that $S(t_{BB})$ were still quite small, but that $S(t_{0})$ were only slightly larger than $S(t_{BB})$ (in comparison with how much bigger the maximal value of S is). Under these conditions, it is easy to confirm that if the universe had only two degrees of freedom, i.e., N=2, then the expected value of the entropy only a single year ago, conditioned on both the values of the entropy at the time of the big bang and its value at $t_{0}$, would be greater than its current value:*

(42) $$\begin{array}{c} E_{P}\left(S\left(t_{t_{0}-1}\right)\;|\;S\left(t_{BB}\right),S\left(t_{0}\right)\right)\;>\;S\left(t_{0}\right) \end{array}$$

*It is not clear how strong this “bump up” of the expected value of the actual thermodynamic entropy in the recent past of the actual physical universe is, where the analogs of both N and L are astronomically larger than two (literally). Presumably, the bump up is not strong enough to overcome the strong “pull” towards lower past entropy values due to the enormous drop between the values of the entropy at the time of the Big Bang and its current ($t_{0}$) value. After all, increase L, causing value $d(t_{0})$ to be vastly larger than $d(t_{BB})$ while still far less then the maximal value. Then, since the entropy scales with d as $O(d^{N-1})$, and since by the Past Hypothesis, $S\left(t_{BB}\right)\ll S\left(t_{0}\right)$, the difference between the expected entropy in the recent past and the current entropy starts to shrink as a move further into the past is realized, presumably ultimately turning over and starting to decrease very sharply, in order to decrease by $S\left(t_{BB}\right)-S\left(t_{0}\right)$ by the time the move $t_{0}-t_{BB}$ years into the past is complete.*

*However, the calculation confirming this has not been conducted, nor has the associated calculation of how far into the past the time is where the expected entropy turns over and starts to decrease the further into the past the move is.*

These arguments imply that the physical phenomenon that Type-3 memory systems rely on would no longer occur in mesoscopic systems, since they do not obey the second law. On the other hand, these arguments also imply that those phenomena underlying Type-3 memory systems will indeed hold if we restrict our attention to macroscopic systems. However, it would be interesting to calculate the precise magnitude of the turnover effect in our physical universe to confirm this supposition.

## 7. Future Work and Open Issues

Finally, we mention two avenues for investigation that the analysis in this paper highlights but does not address.

First, in this paper, we consider three types of memory systems, which are the three types of memory system we can find examples of in the real, physical world. We provide no proof that no other type of memory system is possible. One obvious avenue for future work is to investigate this issue further.

Second, we show how, due to the second law, there can be Type-3 memory systems of the past. We also argue (semi-formally) that the human brain involves such types of memory. Based on our discussion, we consider it plausible that Type-3 memories cannot be of the future. In essence, this is because we do not see a potential mechanism that could play the role the second law of thermodynamics plays in such putative Type-3 memories of the future. But we provide no formal proof that Type-3 memory systems can only be of the past. This issue is thus left for future research.

## Data Availability

No data was created for this research.
